# Cyclin B1 Overexpression Induces Cell Death Independent of Mitotic Arrest

**DOI:** 10.1371/journal.pone.0113283

**Published:** 2014-11-21

**Authors:** Joshua M. Eichhorn, Anisha Kothari, Timothy C. Chambers

**Affiliations:** Department of Biochemistry and Molecular Biology, University of Arkansas for Medical Sciences, Little Rock, Arizona, United States of America; Virginia Commonwealth University, United States of America

## Abstract

Microtubule inhibitors are widely used in cancer chemotherapy. These drugs characteristically induce mitotic arrest and cell death but the mechanisms linking the two are not firmly established. One of the problems is that cancer cells vary widely in their sensitivity to these agents, and thus comparison of data from different systems is difficult. To alleviate this problem we sought to molecularly induce mitotic death and study its mechanisms, by expressing non-degradable cyclin B (R42A) in HeLa cells. However, this approach failed to induce significant mitotic arrest, Cdk1 activation, or phosphorylation of anti-apoptotic Bcl-2 proteins, all characteristics of cells treated with microtubule inhibitors. Furthermore, cyclin B1-R42A induced rapid cell death, and when expressed in synchronized cells, cell death occurred in G1 phase. Decreasing the plasmid concentration reduced transfection efficiency but restored mitotic arrest and eliminated non-specific death. These results show that inappropriate overexpression of cyclin B1 causes non-specific cell death and suggest caution in its use for the study of mitotic events.

## Introduction

The cell cycle is regulated by the sequential and coordinated actions of cyclin-dependent protein kinases (Cdks) and their associated cyclin subunits [1], [2]. As cells approach mitosis, nuclear cyclin B levels rise dramatically which leads to the activation of Cdk1/cyclin B1 complexes. Once activated, Cdk1/cyclin B1 is the major regulator of the transition into and through mitosis. Cdk1 phosphorylates a variety of substrates during mitosis including nuclear lamins and linker histones which are involved in nuclear envelope breakdown and chromatin condensation, respectively [3], [4]. On the other hand, mitotic exit requires inactivation of Cdk1 through the ubiquitination and degradation of cyclin B1 [5], [6]. Expression of a non-degradable form of cyclin B1, which contains a R42A mutation in its destruction box preventing its recognition by the anaphase promoting complex (APC), has been shown to arrest cells in mitosis since Cdk1 activity remains elevated [6]–[8]. As such, expression of non-degradable cyclin B1-R42A represents a molecular approach to induce mitotic arrest in the absence of spindle damage.

Sustained mitotic arrest leads to apoptotic cell death but the signaling pathways that link mitotic arrest and apoptosis are still controversial and not clearly established [9], [10]. Elucidation of these apoptotic pathways is pivotal in understanding the molecular basis of sensitivity and resistance to microtubule inhibitors and other anti-mitotic agents. Studies using microtubule inhibitors such as vinblastine are complicated by the fact that different cell types differ in their sensitivity, and the fate of cells is dependent on drug concentration. These drugs target tubulin or microtubule and suppress microtubule dynamic instability, leading to prolonged activation of the spindle checkpoint and mitotic arrest [10], [11]. Cells with a robust spindle checkpoint may arrest and die in mitosis, but cells with a weakened checkpoint may undergo mitotic slippage and die in interphase or survive [11], [12]. In addition, microtubule inhibitors may target interphase microtubules [13]. In order to avoid these problems, we investigated the use of non-degradable cyclin B1 as a means to induce mitotic arrest and mitotic death in HeLa cells. However, we found to our surprise that its overexpression resulted in non-specific cell death independent of mitotic arrest, in contrast to effects typically observed with vinblastine and other microtubule inhibitors. These results indicate that caution should be exercised when using this approach for the study of mitotic events.

## Materials and Methods

### Materials

Antibodies against Bcl-xL (2762), phospho-histone H3 (P-H3), and glyceraldehyde-3-phosphate dehydrogenase (GAPDH) were purchased from Cell Signaling (Beverly, MA); antibodies against cyclin B1 (sc-245), Bcl-2 (sc-509), and Mcl-1 (M22) were purchased from Santa Cruz (Santa Cruz, CA); antibody for Poly(ADP-ribose) polymerase (PARP) was purchased from BD Biosciences (San Jose CA). Vinblastine and histone H1 was purchased from Sigma Aldrich (St. Louis, MO) and thymidine was purchased from EMD Biosciences (Gibbstown, NJ). Cell death analysis was measured using a Cell Death Detection ELISA kit from Roche (Penzberg, Germany). Propidium iodide/RNAse staining buffer used for cell cycle analysis was purchased from BD Biosciences (San Jose, CA).

### Cell culture, preparation of whole cell extracts, and immunoblotting

HeLa human cervical carcinoma cell line was maintained in monolayer culture at 37°C and 5% CO_2_ in Dulbecco's modified Eagle's medium (DMEM) supplemented with 10% fetal bovine serum, 2 mM L-glutamine, 50 units/mL penicillin, and 50 µg/mL streptomycin. Cells were synchronized at the G1/S boundary by double thymidine block as described previously [14]. Whole cell extracts for kinase assays and immunoblot analysis were prepared as described previously [15], and protein concentration was determined using a Bradford assay (Bio-Rad, Hercules, CA) according to manufacturer's instructions.

### Transfection

Plasmids encoding GFP (6.2 kb) and cyclin B1-GFP (R42A or wild-type) (7.5 kb), cloned into the pcDNA3.1 vector, were kindly provided by Dr. Jonathon Pines, University of Cambridge, UK. DNA transfections were performed using Lipofectamine 2000 (Invitrogen, Carlsbad, CA) in Opti-MEM according to manufacturer's instructions. Transient transfections were performed using 2 µg plasmid DNA, unless otherwise indicated, with 10 µl Lipofectamine 2000 reagent. Fresh media containing serum was added 6 h post-transfection.

### Cell cycle analysis

After transfection, HeLa cells were harvested and resuspended in DMEM at a concentration of 10^6^ cells/ml. Cell cycle analysis was performed using propidium iodide staining and flow cytometry according to the manufacturer's instructions (BD Pharmigen) by the UAMS Flow Cytometry Core Facility using a FACSCalibur (Becton Dickinson, Mountain View, CA). The data were analyzed using the ModFit DNA analysis program (Verity Software House).

### Cdk1 kinase assay

Cdk1 activity was determined as described previously [15]. Briefly, whole cell extracts containing 5 µg protein were incubated with H1 histone (5 µg) in a reaction mixture containing 25 mM Tris-HCl pH 7.5, 10 mM MgCl_2_, 5 mM DTT, 1 µM ATP, and 1 µCi [γ^32^P]ATP at 30°C for 20 min. The reaction was stopped by the addition of EDTA to 20 mM, acidified by the addition of acetic acid to 15% v/v, and spotted onto 2 cm P81 phosphocellulose filter discs (Fisher) which were subsequently washed in 75 mM phosphoric acid for measurement of ^32^P incorporation by scintillation counting. Background activity, obtained in reactions incubated in the absence of substrate, was subtracted.

### Apoptosis assay

Apoptosis was determined using a Cell Death ELISA assay, which detects formation of soluble nucleosomes upon DNA fragmentation, according to manufacturer's instructions (Roche Applied Science). Nucleosome formation was quantified by absorbance at 405 nm.

### Statistical analysis

Data were analyzed using Student's t-test with p≤0.05 considered significantly different. Assays were conducted with replicates of 3 and all experiments repeated at least once with essentially identical data.

## Results

### Expression of non-degradable cyclin B1 fails to recapitulate events induced by microtubule inhibitors

**Figure 1:**
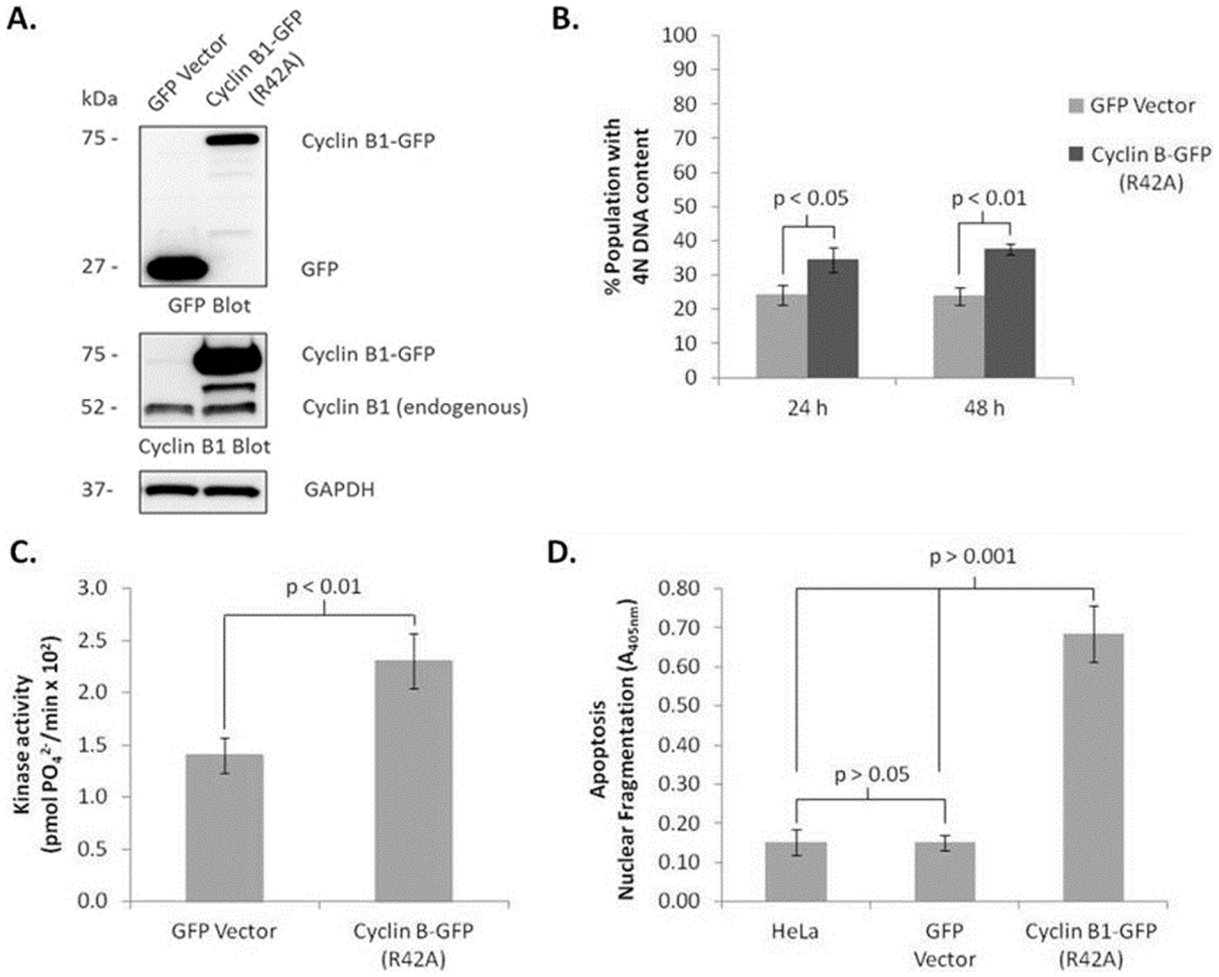
Expression of non-degradable cyclin B1 leads to a modest increase in mitotic arrest and activation of Cdk1, and rapid apoptosis. HeLa cells were transfected with 2 µg plasmid DNA encoding GFP vector or encoding non-degradable cyclin B1-R42A-GFP for the times indicated and subjected to: **A.** Immunoblotting for GFP and cyclin B1. GAPDH was used as a loading control. Apparent molecular weights of the proteins are indicated on the left. **B.** Cell cycle analysis by propidium iodide staining and flow cytometry. Mitotic arrest is indicated by an increase in the percentage of cells with 4N DNA content. **C.** Cdk1 assay performed as described in Materials and Methods. ^32^P incorporation was determined by scintillation counting. Values shown have been corrected for background kinase activity (no substrate) and represent the mean ± standard deviation (n = 3). **D.** Cell death ELISA, as described in Materials and Methods. Untransfected HeLa cells were used as a negative control. Results given are mean ± standard deviation (n = 3).

**Figure 4:**
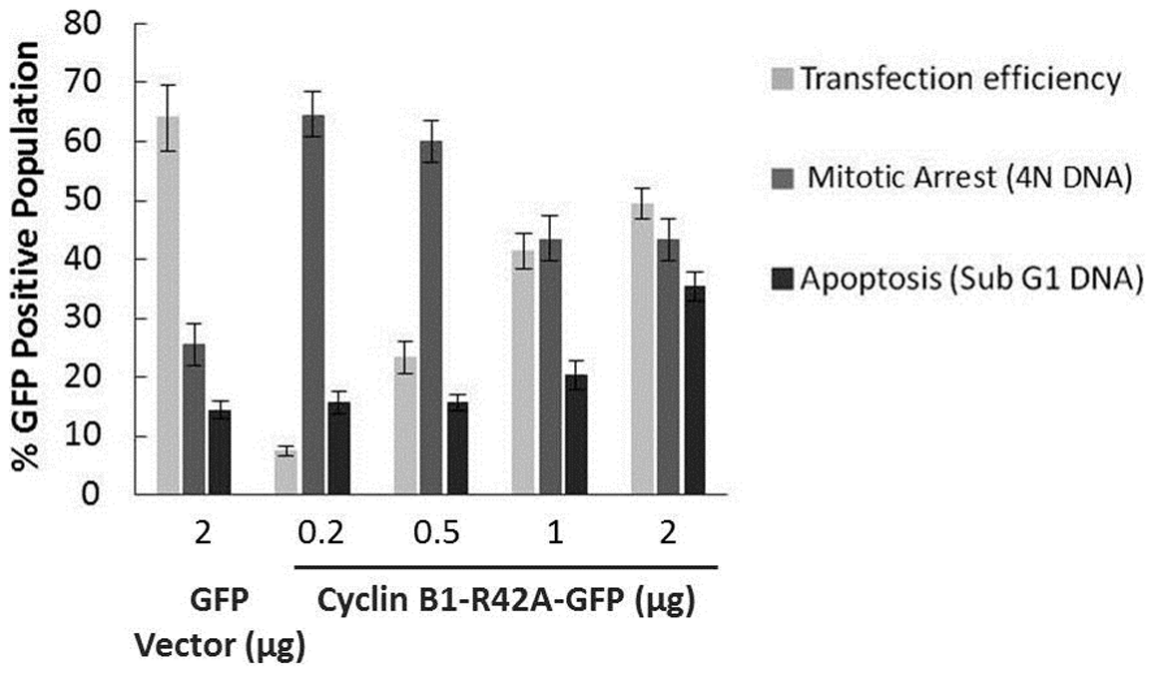
Dose-dependent effects of cyclin B1-R42A-GFP expression on extent of mitotic arrest and cell death. HeLa cells were transfected with increasing concentrations (0.2–2 µg) of plasmid encoding cyclin B1-R42A-GFP or 2 µg plasmid encoding GFP vector, as indicated, for 24 h and then subjected to propidium iodide staining and analyzed by flow cytometry. The proportion of GFP positive cells was used to assess transfection efficiency. The DNA content of GFP positive cells was used to determine the percentage of transfected cells undergoing mitotic arrest (4N DNA) or apoptosis (sub-G1 DNA). Data shown are mean ± standard deviation of three independent experiments.

HeLa cells were transiently transfected with plasmids encoding either GFP-tagged non-degradable cyclin B1 (cyclin B1-R42A-GFP) or GFP vector, and expression verified by immunoblotting using both GFP and cyclin B1 antibodies (Fig. 1A). Based on immunoreactivity with the GFP antibody, and assuming both proteins are recognized with similar affinity, GFP at 27 kDa was expressed at a higher relative level than cyclin B1-R42A-GFP at 75 kDa. This may reflect in part different expression efficiencies, different turnover rates, and/or be related to the fact that GFP overexpression was generally well tolerated by the cells, whereas cyclin B1 overexpression was not and caused cell death, as described below. Strong cyclin B1 immunoreactivity was observed at 75 kDa after transfection with cyclin B1-R42A-GFP, which was absent after transfection with GFP, as expected (Fig. 1A). Endogenous cyclin B1 levels (at 52 kDa) were similar for GFP- or cyclin B1-R42A-GFP-tranfected cells, and the intermediate band seen with the latter is likely a minor degradation product. Expression of cyclin B1-R42A-GFP caused an increase in cells with 4N DNA content to about 35% from about 23% in control cells expressing GFP vector (Fig. 1B), under conditions where transfection efficiency was about 50% (see Fig. 4). Cells with 4N DNA content are those in G2 or M phase, and an increase in this population typically reflects mitotic arrest. We did not observe evidence of arrest at other cell cycle phases. Taking into account transfection efficiency, the increase in cells with 4N DNA content is approximately two-fold. In contrast, previous work has indicated after 24 h exposure to the microtubule inhibitor vinblastine, 94% of HeLa cells have 4N DNA content, representing an increase of over 4-fold relative to control cells. [16]. A kinase assay was performed as described in Materials and Methods to determine whether expression of cyclin B1-R42A-GFP increased Cdk1 activity. A marginal yet significant increase in Cdk1 activity of less than two-fold was observed (Fig. 1C). This compares to a 10- to 15-fold increase in Cdk1 activity obtained with vinblastine treatment [15]. Taken together, the results of Fig. 1A–C indicate that non-degradable cyclin B1 induces an incomplete mitotic arrest. Expression of cyclin B1-R42A-GFP resulted in induction of apoptosis at 24 h post-transfection, as determined by nuclear fragmentation as described in Materials and Methods (Fig. 1D). Previously it has been shown that HeLa cells treated with microtubule inhibitors initiate apoptosis after prolonged mitotic arrest, with the major increase occurring 24–48 h post-treatment [16], [17]. Overall, the results of Fig. 1 indicate that overexpression of cyclin B1 fails to faithfully recapitulate the events, namely robust mitotic arrest and subsequent cell death, typically observed after treatment with microtubule inhibitors.

**Figure 2:**
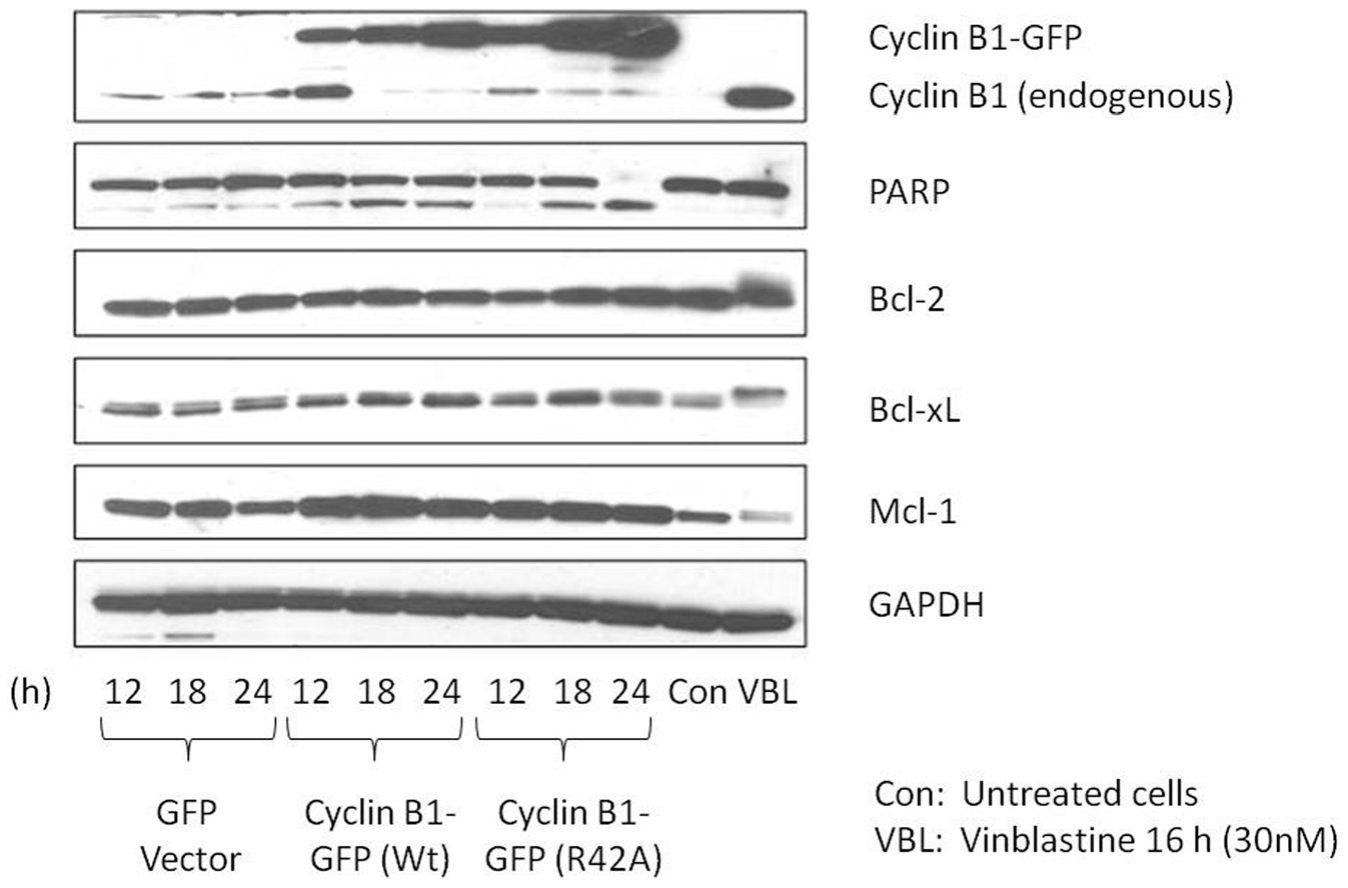
Cyclin B1 overexpression fails to recapitulate effects of vinblastine on phosphorylation of anti-apoptotic Bcl-2 proteins. HeLa cells were transfected with 2 µg plasmids encoding either wild-type cyclin B1-GFP (Wt), non-degradable cyclin B1-R42A-GFP, or GFP vector for 12–24 h. Untreated untransfected or vinblastine treated (30 nM, 24 h) HeLa cells are shown in the right two lanes. Extracts were prepared and subjected to immunoblotting for the proteins indicated. GAPDH was used as a loading control. Apparent molecular weights (in kDa) are indicated.

Microtubule inhibitors characteristically induce phosphorylation and subsequent inactivation of anti-apoptotic Bcl-2 proteins [18]. To determine if non-degradable cyclin B1 acted similarly, HeLa cells were transiently transfected with plasmids encoding cyclin B1-R42A-GFP or GFP vector, for 12, 18 or 24 h. We also used a plasmid encoding wild-type cyclin B1-GFP in order to compare its effects to the non-degradable form. The shorter transfection times were chosen due to the significant amount of nuclear fragmentation and apoptosis occurring by 24 h (Fig. 1D). Immunoblot analysis showed that the level of expression of cyclin B1-R42A-GFP increased with time of transfection as anticipated (Fig. 2). PARP cleavage, a measure of caspase-3 activation and apoptosis induction, was evident at 18 h and nearly complete at 24 h. During this time period there was little increase in endogenous cyclin B1 and no evidence of phosphorylation of anti-apoptotic Bcl-2 proteins (Bcl-2, Bcl-xL, and Mcl-1), indicated by a lack of band mobility shift. Very similar results were obtained after transfection with wild-type cyclin B1-GFP, although PARP cleavage was incomplete, possibly resulting from reduced expression of cyclin B1-GFP compared to that obtained with the non-degradable form (Fig. 2). Expression of native GFP had no observable effect on these parameters under these conditions. In contrast, after vinblastine treatment, endogenous cyclin B1 increased, phosphorylation of anti-apoptotic Bcl-2 proteins was observed, and PARP remained intact until 24 h when cleavage was observed to be initiated. The results of Figs. 1 and 2 suggest that overexpression of cyclin B1 induces non-specific death in the absence of mitotic arrest.

### Expression of cyclin B1-GFP leads to non-mitotic cell death

**Figure 3:**
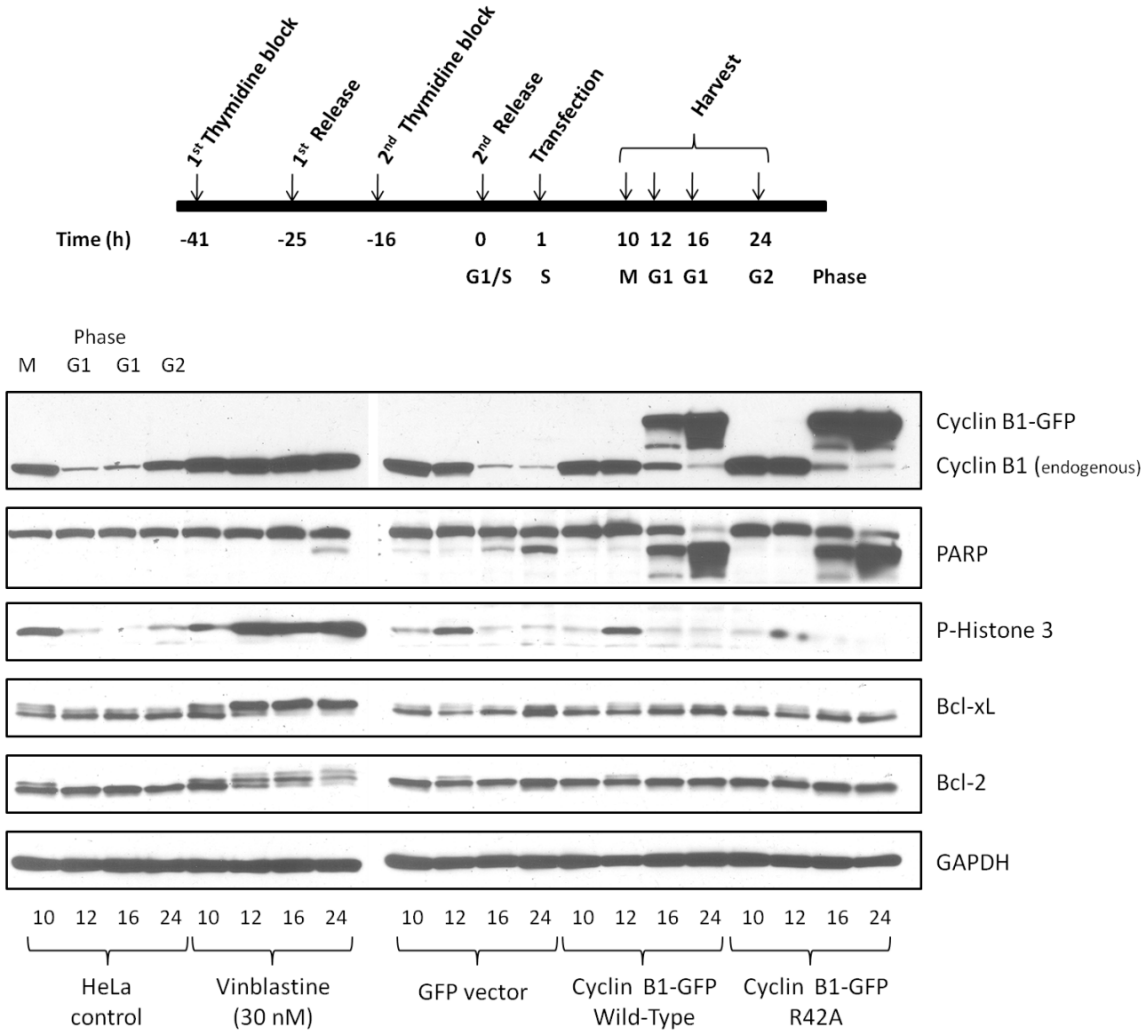
Overexpression of cyclin B1 in synchronized cells induces cell death in G1 phase independent of mitotic arrest. HeLa cells were synchronized at the G1/S boundary using double thymidine block, released into fresh media, and 1 h post-release were untreated (vehicle), treated with 30 nM vinblastine, or transfected with 2 µg plasmids encoding either GFP vector, wild-type cyclin B1-GFP or cylcin B1-R42A-GFP. Cells were harvested at intervals 10–24 h post-release and extracts subjected to immunoblotting for the indicated proteins. Top panel, experimental scheme, with corresponding cell cycle phases indicated. Bottom panel, immunoblots.

In order to confirm that overexpression of cyclin B1 resulted in non-mitotic death, HeLa cells were synchronized at the G1/S boundary by double thymidine block, released into fresh media, and transfected with plasmids encoding cyclin B1-R42A-GFP, wild-type cyclin B1-GFP, or GFP vector. Cells were harvested 10–24 h post-release and immunoblot analysis was performed to monitor expression of exogenous cyclin B1-GFP as well as mitotic markers (endogenous cyclin B1 and phospho-histone 3, P-H3) and PARP cleavage as an indicator of apoptosis (Fig. 3). Previous studies have shown that at 10 h post-release cells are in M phase, and at 12–16 h in G1 phase and at 24 h in G2 phase of the subsequent cell cycle [14], [15], [17]. Release of cells into DMSO vehicle (Fig. 3) confirmed this sequence, as endogenous cyclin B1 and P-H3 levels were relatively high at 10 h and subsequently decreased as cells exited M phase into G1 phase at 12–16 h. In contrast when released in the presence of vinblastine, endogenous cyclin B1 and P-H3 levels became elevated and sustained, Bcl-2 and Bcl-xL became phosphorylated, and PARP cleavage initiated at 24 h, emphasizing the requirement for prolonged mitotic arrest prior to apoptosis induction. When synchronized cells were transfected with GFP vector, results similar to DMSO were obtained, except that mitotic entry and exit appeared to be delayed slightly, and there was evidence of PARP cleavage at 24 h. However, the extent of PARP cleavage was relatively minor, and may reflect an artifact of the transfection process, as we have observed similar effects when introducing other types of DNA via transfection into HeLa cells. When synchronized cells were transfected with wild-type cyclin B1-GFP or cyclin B1-R42A-GFP, expression of cyclin B1-GFP was observed as early as 16 h post-release. This coincided with cells being in G1 phase, as evidenced by diminished endogenous cyclin B1 and P-H3 levels. Remarkably, PARP cleavage was initiated at this same time point, upon expression of the plasmid-encoded proteins when cells were in G1 phase, with near complete PARP cleavage by 24 h.

### Cyclin B1 expression level is critical

These results show that expression of high levels of cyclin B1-GFP leads to non-specific non-mitotic death. We therefore sought to determine if a threshold existed where lower levels of cyclin B1-GFP would be effective in inducing mitotic arrest without causing non-specific cytotoxic effects. HeLa cells were transfected with increasing amounts of plasmid (0.2–2 µg) encoding cyclin B1-R42A-GFP, followed by staining with propidium iodide and analysis by flow cytometry for transfection efficiency (% GFP positive cells), as well as quantification of the number of transfected cells undergoing mitotic arrest (4N DNA) and apoptosis (sub-G1 DNA) (Fig. 4). Transfection efficiency was dependent on plasmid concentration; transfection efficiency was 7% with 0.2 µg of plasmid compared to nearly 50% with 2 µg of plasmid (Fig. 4). With 0.2 µg of cyclin-R42A-GFP plasmid DNA, 65% of GFP-positive cells were found to contain 4N DNA content and a relatively small percentage contained sub-G1 DNA content (15%). However, with 2 µg of cyclin-R42A-GFP plasmid DNA, the higher transfection efficiency was accompanied by an increase in the number of GFP-positive cells with sub-G1 DNA content (35%) and a lower percentage of cells with 4N DNA content (45%) at 24 h post-transfection when compared with cells transfected with lower amounts of plasmid DNA (Fig. 4). These values differed markedly from those obtained after transfection with 2 µg of GFP vector DNA, which was transfected efficiently but was without effect on these parameters (Fig. 4). Thus low levels of plasmid induced mitotic arrest without overt cell death whereas high levels of plasmid were less effective in inducing mitotic arrest and promoted cell death Note that the proportion of cells with 4N DNA content after transfection with 2 µg cyclin B1-GFP plasmid (45%) in Fig. 4 is greater than the value of 35% observed in Fig. 1B because in Fig. 4 only the GFP-positive cells were quantified, whereas in Fig. 1B the total population was examined. Taken together, the results of Fig. 4 indicate that there exists a delicate balance between transfection efficiency and non-specific cell death caused by significant overexpression of cyclin B1-GFP. Representative flow cytometry histograms of untransfected, GFP vector-transfected, or cyclin B1-R42A-GFP-transfected, at 0.2 and 1.0 µg, are shown in Figure S1.

## Discussion

It is well established that Cdk1/cyclin B1 is essential for entry into and progression through mitosis and thus activation of this complex plays a key regulatory role in cell proliferation. Importantly, sustained and inappropriate activation of Cdk1/cyclin B1 plays an opposite role, and mediates pro-apoptotic signaling in response to mitotic arrest [15]. This is due, at least in part, to the phosphorylation and inactivation of anti-apoptotic Bcl-2 proteins mediated by Cdk1/cyclin B1 [15]. However, Cdk1/cyclin B1 also phosphorylates and inactivates other substrates such as caspase-9, which protects cells from apoptosis during mitotic arrest [19]. Precisely how these pro-apoptotic and pro-survival functions are regulated and integrated remains to be established. Mitotic arrest-induced cell death pathways are most commonly studied using microtubule inhibitors or other anti-mitotic agents. However, the use of drugs is complicated by several confounding variables. For example, the sensitivity of different cell lines to microtubule inhibitors varies considerably, and there exists drug-specific effects and concentration-dependent responses [10]. In addition, microtubule inhibitors may also interfere with interphase processes [13], and they activate diverse signaling pathways [20]. Expression of non-degradable cyclin B1 has previously been shown to result in sustained activation of Cdk1 and mitotic arrest [6]–[8]. In this study we investigated this approach as a means to induce mitotic arrest in order to study the molecular mechanisms of mitotic death in the absence of treatment with microtubule inhibitors.

While expression of cyclin B1-R42A-GFP led to an increase in Cdk1 activity and in the proportion of cells with 4N DNA content, the magnitude of these changes was much smaller than we had observed previously with microtubule inhibitors. In addition, overexpression of cyclin B1 led to rapid cell death in the absence of typical markers of mitotic death, such as elevated levels of endogenous cyclin B1 and phospho-H3, as well as phosphorylation of anti-apoptotic Bcl-2 proteins. Furthermore, the use of synchronized cells provided strong evidence that cell death can be initiated in G1 phase, quite independent of mitosis. When similar amounts of cyclin B1-R42A-GFP or wild-type cyclin B1 were transfected into HeLa cells, the expression level of the former, determined by immunoblotting for cyclin B1, was higher (Figs. 2 and 3). This was presumably due to the resistance to degradation of the mutant form. Nonetheless, overall similar results were obtained with both plasmids, suggesting that overexpression of cyclin B1 per se, rather than specifically the mutant form, is detrimental to the cell. This is consistent with the finding that lower amounts of plasmid DNA led to mitotic arrest without rapid non-specific death, although this came at the expense of transfection efficiency (Fig. 4). Why excessive production of cyclin B1 drives apoptotic death independent of the cell cycle is presently unclear.

Overall, our findings indicate that caution should be exercised when using cyclin B1 overexpression as an experimental strategy to study mitotic events, especially mitotic death. Optimization of the plasmid concentration and careful evaluation of the effects occurring are necessary to avoid problems which could result in data misinterpretation. Performing a low level transfection followed by sorting using FACS analysis to recover the cells which express the protein would be desirable. An alternative approach would be to stably transfect the cells with plasmid encoding non-degradable cyclin B1 under control of an inducible promoter [12]. This would enable controlled expression and if used in conjunction with cell synchronization allow expression at appropriate points in the cell. Finally, knockdown of the Cdc20, an activator of the APC, has been used successfully to induce mitotic arrest, and could be considered as an attractive and alternate strategy [21].

## Supporting Information

Figure S1 **Dose-dependent effects of cyclin B1-R42A-GFP expression on transfection efficiency, mitotic arrest and cell death.** Representative flow cytometry histograms of HeLa cells transfected with plasmid encoding cyclin B1-R42A-GFP or plasmid encoding GFP vector (1 µg), as indicated, for 24 h and then subjected to propidium iodide staining. The proportion of GFP positive cells (FL1-H) was used to assess transfection efficiency. The DNA content (FL2-A) of GFP positive cells was used to determine the percentage of transfected cells undergoing mitotic arrest (4N DNA) or apoptosis (sub-G1 DNA). Non-transfected HeLa cells were used as a negative control to establish proper gating for GFP positive cells.
(TIF)
