# Correction: Cyclin B1 Overexpression Induces Cell Death Independent of Mitotic Arrest

**DOI:** 10.1371/journal.pone.0117624

**Published:** 2015-02-06

**Authors:** The PLOS ONE Staff

The state listed in the affiliation for all authors is incorrect. The publisher apologizes for this error. The correct affiliation is: Department of Biochemistry and Molecular Biology, University of Arkansas for Medical Sciences, Little Rock, Arkansas, United States of America
