# Supplementary figures and images for: Cyclin B1 Overexpression Induces Cell Death Independent of Mitotic Arrest
Source: PLoS One. 2014 Nov 21;9(11):e113283. doi: 10.1371/journal.pone.0113283 (PMC4240608; doi:10.1371/journal.pone.0113283)

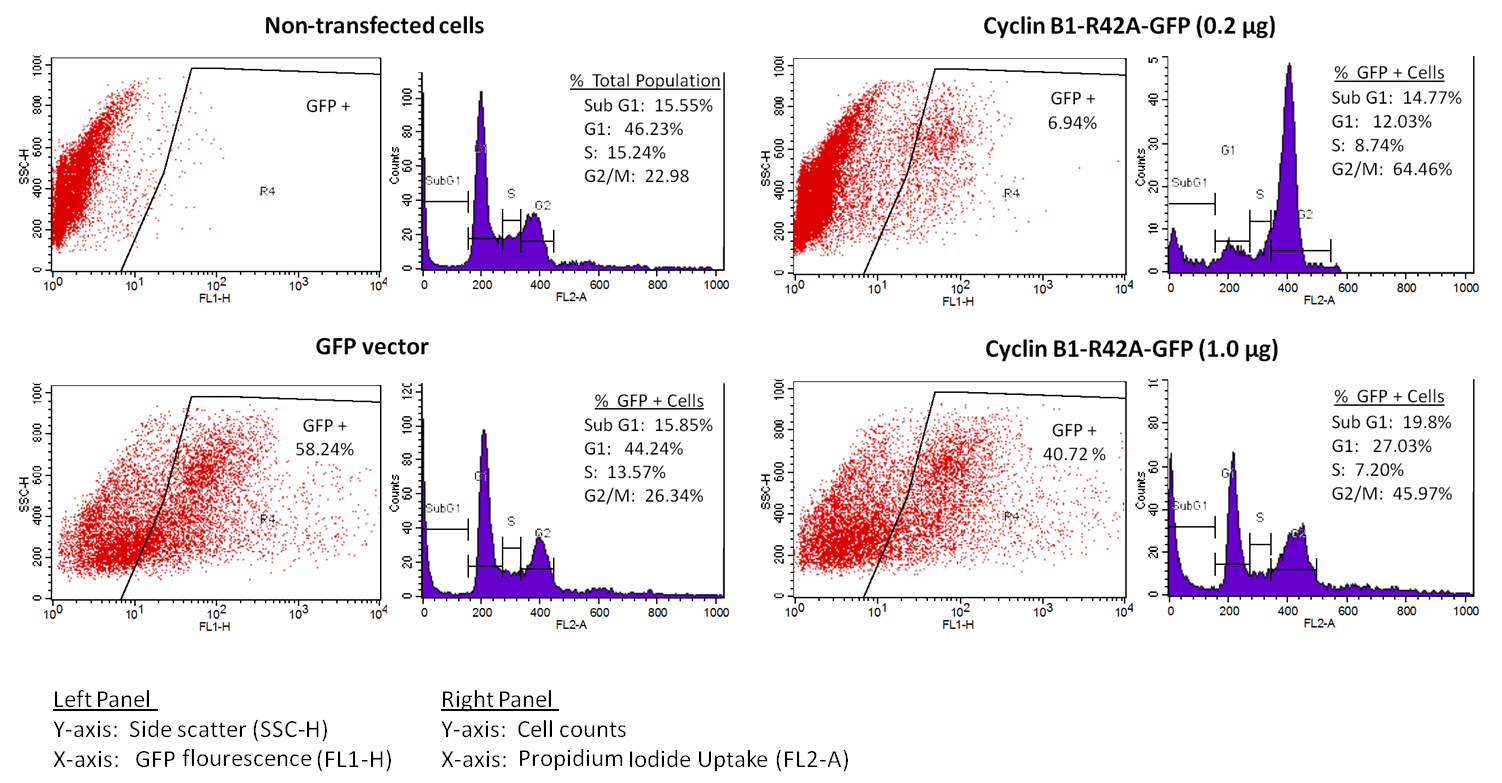

Supplement: Figure S1 — Dose-dependent effects of cyclin B1-R42A-GFP expression on transfection efficiency, mitotic arrest and cell death. Representative flow cytometry histograms of HeLa cells transfected with plasmid encoding cyclin B1-R42A-GFP or plasmid encoding GFP vector (1 µg), as indicated, for 24 h and then subjected to propidium iodide staining. The proportion of GFP positive cells (FL1-H) was used to assess transfection efficiency. The DNA content (FL2-A) of GFP positive cells was used to determine the percentage of transfected cells undergoing mitotic arrest (4N DNA) or apoptosis (sub-G1 DNA). Non-transfected HeLa cells were used as a negative control to establish proper gating for GFP positive cells. (TIF) [file pone.0113283.s001.tif]
